# Conservation of the glucan phosphatase laforin is linked to rates of molecular evolution and the glucan metabolism of the organism

**DOI:** 10.1186/1471-2148-9-138

**Published:** 2009-06-22

**Authors:** Matthew S Gentry, Rachel M Pace

**Affiliations:** 1 Department of Molecular and Cellular Biochemistry, University of Kentucky College of Medicine, 741 S. Limestone, BBSRB, B177, Lexington, Kentucky 40536-0509, USA

## Abstract

**Background:**

Lafora disease (LD) is a fatal autosomal recessive neurodegenerative disease. A hallmark of LD is cytoplasmic accumulation of insoluble glucans, called Lafora bodies (LBs). Mutations in the gene encoding the phosphatase laforin account for ~50% of LD cases, and this gene is conserved in all vertebrates. We recently demonstrated that laforin is the founding member of a unique class of phosphatases that dephosphorylate glucans.

**Results:**

Herein, we identify laforin orthologs in a protist and two invertebrate genomes, and report that laforin is absent in the vast majority of protozoan genomes and it is lacking in all other invertebrate genomes sequenced to date. We biochemically characterized recombinant proteins from the sea anemone *Nematostella vectensis *and the amphioxus *Branchiostoma floridae *to demonstrate that they are laforin orthologs. We demonstrate that the laforin gene has a unique evolutionary lineage; it is conserved in all vertebrates, a subclass of protists that metabolize insoluble glucans resembling LBs, and two invertebrates. We analyzed the intron-exon boundaries of the laforin genes in each organism and determine, based on recently published reports describing rates of molecular evolution in *Branchiostoma *and *Nematostella*, that the conservation of laforin is linked to the molecular rate of evolution and the glucan metabolism of an organism.

**Conclusion:**

Our results alter the existing view of glucan phosphorylation/dephosphorylation and strongly suggest that glucan phosphorylation is a multi-Kingdom regulatory mechanism, encompassing at least some invertebrates. These results establish boundaries concerning which organisms contain laforin. Laforin is conserved in all vertebrates, it has been lost in the vast majority of lower organisms, and yet it is an ancient gene that is conserved in a subset of protists and invertebrates that have undergone slower rates of molecular evolution and/or metabolize a carbohydrate similar to LBs. Thus, the laforin gene holds a unique place in evolutionary biology and has yielded insights into glucan metabolism and the molecular etiology of Lafora disease.

## Background

Lafora disease (LD; OMIM 254780) is an autosomal recessive neurodegenerative disorder. It is one of five major progressive myoclonus epilepsies (PMEs) [1]. LD commonly presents as a single seizure in the second decade of the patient's life, followed by progressive central nervous system degeneration, intellectual decline, and death within ten years of the first seizure [2-4]. LD is unique among the PMEs because of the patient's rapid neurological deterioration and the accumulation of insoluble glucans/carbohydrates called Lafora bodies (LB) [5,6].

While animals normally store glucans as soluble glycogen, LBs are accumulations of poorly branched, hyperphosphorylated, insoluble glucans and are not glycogen. Forty years ago, Sakai and co-workers biochemically characterized LBs and found that they more closely resemble plant starch than glycogen [6-8]. Although LBs are found in the cytoplasm of most cells, cell death only occurs in neurons [3]. LD patients exhibit increased neuronal cell death, number of seizures, and LB accumulation as they age; thus, it is hypothesized that LBs trigger these symptoms and ultimately the death of the patient [6].

**Figure 1:**
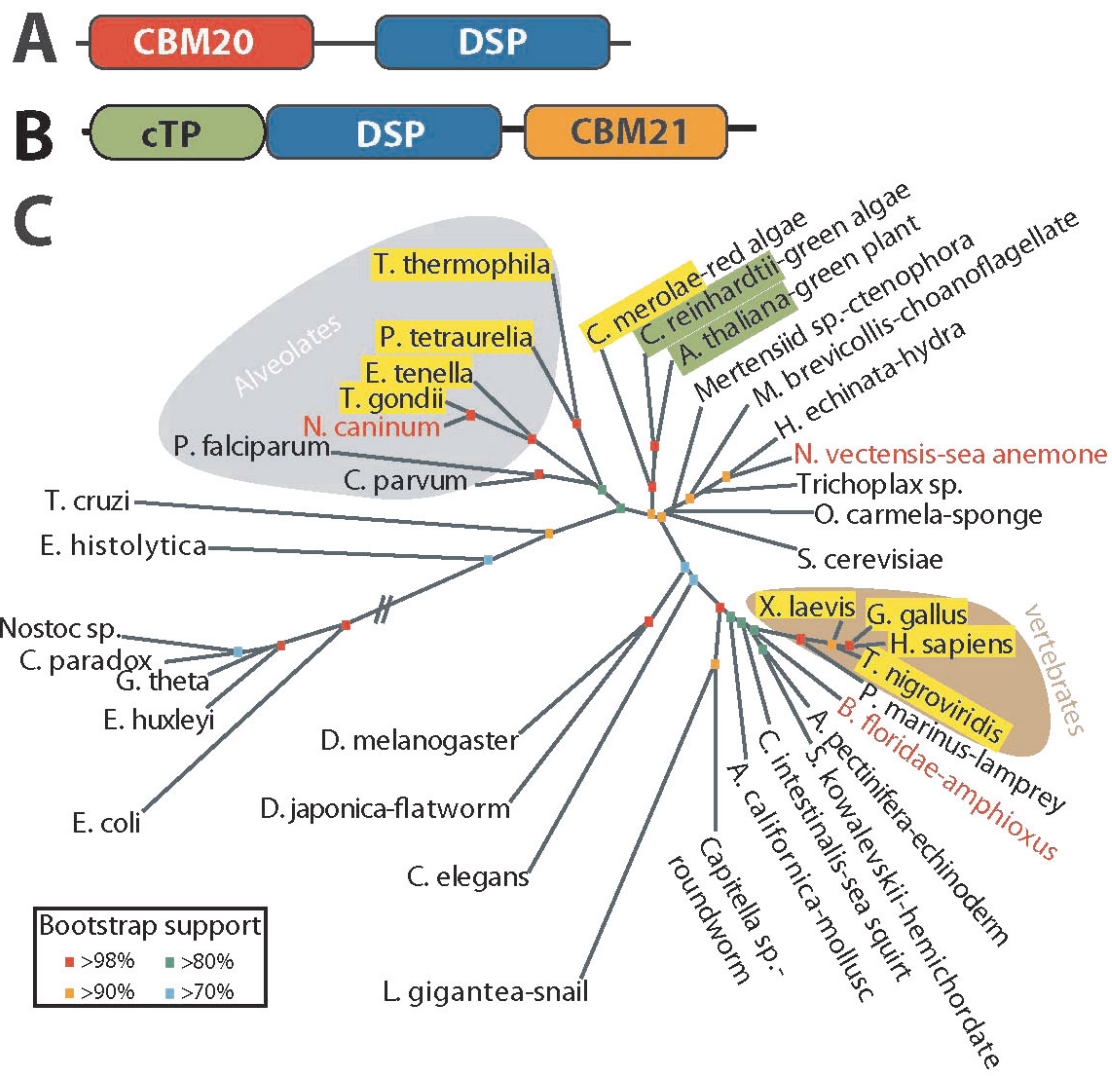
**Domain architecture and orthologs of laforin and SEX4**. *A*, Laforin contains a carbohydrate binding module-family 20 (CBM20) and a dual specificity phosphatase (DSP) domain. *B*, SEX4 is composed of a chloroplast targeting peptide (cTP), dual specificity phosphatase (DSP) domain, and a carbohydrate binding module-family 21 (CBM21). *C*, Unrooted phylogeny of the small subunit ribosomal RNA (SSU rRNA) sequences was generated as described in Methods, and accession numbers are listed in Additional File 5. Organisms containing laforin are boxed in yellow and those containing SEX4 are boxed in green. Organisms in red lettering are the organisms identified in this study that contain a laforin ortholog. Alveolates are shaded with a gray background and vertebrates with a brown background. Bootstrap values are indicated by color coding as indicated in the inset.

*EPM2A *(epilepsy of progressive myoclonus type 2) is mutated in ~50% LD cases [9,10]. *EPM2A *encodes a protein named laforin that contains a carbohydrate binding module (CBM) followed by the canonical dual specificity phosphatase (DSP) active site motif, HCXXGXXRS/T (Cx_5_R) [9,11] (Fig. 1A). Accordingly, recombinant laforin binds glucans, *in vitro *and *in vivo*, and it possesses phosphatase activity *in vitro *[12,13]. Out of ~130 human phosphatases laforin is the only phosphatase with a CBM. While data placed laforin in the context of being intimately, if not directly, involved in glycogen metabolism, the molecular etiology of LD was unknown for almost 100 years.

We recently demonstrated that laforin is not restricted to vertebrate genomes, as originally thought [14], but that laforin orthologs are present in five protists (i.e. single-cell eukaryotes) [15]. Each of these five protists undergoes hibernation during their life cycle and when they hibernate they generate an insoluble glucan as an energy source [16-18]. We recognized that the glucan produced by these protists are biochemically similar to Lafora bodies and proposed that laforin functions to convert insoluble glucans into energy, and in vertebrates it functions to inhibit insoluble glucan accumulation (i.e. LBs) [15].

To test this hypothesis, we utilized a recently characterized gene in *Arabidopsis *called *starch excess 4 (SEX4) *[19,20]. *SEX4 *encodes a protein with similar domains as laforin but in the opposite orientation (Fig. 1B). Disruption of *SEX4 *leads to increased accumulation of insoluble glucans (i.e. starch), a cellular phenotype reminiscent of LD patients [19]. We found that when laforin was targeted to the chloroplast of *sex4*-deficient plants that it rescued the *sex4 *mutant phenotype. Demonstrating that laforin and SEX4 are functional equivalents and that a laforin-like activity is required to regulate the metabolism of insoluble glucans in multiple Kingdoms [15].

We also demonstrated the nature of this activity; we showed that laforin and SEX4 dephosphorylate the glucan itself [15,21,22]. Thus, we proposed that when laforin is absent, phosphates accumulate in glycogen precursors, branching is inhibited, and Lafora bodies form. This prediction was supported by work published 40 years ago showing LBs from human patients are poorly branched and contain 4–5 fold more phosphate than glycogen, and further corroborated by data from the Roach lab confirming these results in a LD mouse model [23,24]. Therefore, laforin regulates an overlooked aspect of glycogen metabolism in vertebrates by removing phosphate from glycans during glycogen synthesis.

Herein, we establish definitive boundaries concerning the evolutionary conservation of laforin by probing more than 210 eukaryotic genomes. We utilized criteria that we previously defined to correctly predict a laforin ortholog in the genome of the protozoan *Neospora caninum*. In addition, we extend our previous results by uncovering putative laforin orthologs in two invertebrates, *Nematostella vectensis *and *Branchiostoma floridae*. We cloned the respective genes and biochemically verified that they are laforin orthologs. Furthermore, we present evidence and hypothesize why laforin is conserved in these two invertebrates and is absent in all others sequenced to date. Cumulatively, these results demonstrate that the glucan phosphatase laforin is conserved in a subset of eukaryotic organisms from an array of evolutionary niches, vertebrates, invertebrates, and protists, and highlights the fundamental importance of glucan phosphorylation/dephosphorylation.

## Results and discussion

Carbohydrate binding modules (CBMs) are domains typically found in glucosylhydrolases and glucotransferases in bacterial, fungal, or plant genomes [25-27]. The vast majority of enzymes containing CBMs utilize the domain to bind a specific glucan and enzymatically act on the sugar, as in the case of α-amylase [25]. Accordingly, we recently demonstrated, and others confirmed, that laforin and SEX4 bind and dephosphorylate glucans, glycogen and starch, respectively [15,21,23,24]. While laforin and SEX4 bind similar types of glucans, they utilize evolutionarily distinct CBMs [25]. CBMs are classified into fifty-three evolutionarily distinct families, based on primary sequence, secondary and tertiary predictions, and crystal structures [25]. Laforin contains an amino-terminal CBM20 and SEX4 a carboxy-terminal CBM21 type CBM (Fig. 1A &1B). Although laforin and SEX4 have evolutionarily distinct CBMs, multiple groups have proposed that CBM20 and CBM21 may share a common evolutionary origin and suggest keeping distinct families grouped into a common CBM clan [28-31].

### Confirmation of laforin predictions

Previously we reported that out of 170 eukaryotic genomes (including 94 protozoan) and 670 bacterial genomes that the laforin gene is only conserved in vertebrate genomes and in five protozoan genomes, *Toxoplasma gondii*, *Eimeria tenella*, *Tetrahymena thermophila*, *Paramecium tetraurelia*, and *Cyanidioschyzon merolae *[15] (Fig. 1C). In addition, we demonstrated that *SEX4 *is conserved in green algae and land plants (collectively known as Archaeplastida/Kingdom Plantae) and showed that while laforin and SEX4 are not orthologous proteins that they are functional equivalents [15] (Fig. 1C). Our phylogenetic analyses and examination of the biology and evolution of the five protists that have laforin led us to propose three criteria to predict if a protozoan genome would contain laforin: the organism must 1) be of red algal descent, 2) possess a true mitochondrion, and 3) produce an insoluble glucan (e.g. floridean starch, amylopectin granules, etc.) [15]. Out of the 170 organisms that we probed, we found that if an organism lacked one of these qualities then it lacked laforin and if it possessed all of these qualities then it possessed laforin [15]. Therefore, *Plasmodium *species lack laforin because they do not metabolize insoluble glucans, *Cryptosporidium *species lack laforin because they lack true mitochondria, and *Chlamydomonas *lack laforin because they are not of red-algal descent (and instead have SEX4) [15]. Some of the genomes that we originally probed were incomplete at the time. However, based on the above three criteria, we postulated that the following four organisms currently being sequenced would contain laforin, *Galdieria sulphuraria, Guillardia theta*, *Neospora caninum*, and *Sarcocystis neurona *[15].

In light of the relatedness between CBM20 and CBM21 CBMs, we probed 122 protozoan genomes (28 more than previously) searching for a protein containing a CBM20 or CBM21 domain followed by a phosphatase domain. In order to enhance our likelihood of uncovering a laforin ortholog, we performed BLASTp and tBLASTn searches of multiple databases (Additional File 1) using human (Hs-) and *C. merolae *(Cm-) laforin, as *C. merolae *is likely the most evolutionarily ancient organism with laforin and Cm-laforin was the least identical (25%) to Hs-laforin of all the protozoan laforin orthologs previously identified [15]. To ensure that we did not miss a laforin-like protein in these genomes, we also searched the same databases using the same search methods for proteins with a DSP domain followed by a CBM20 or CBM21, i.e. SEX4 orthologs.

**Figure 2:**
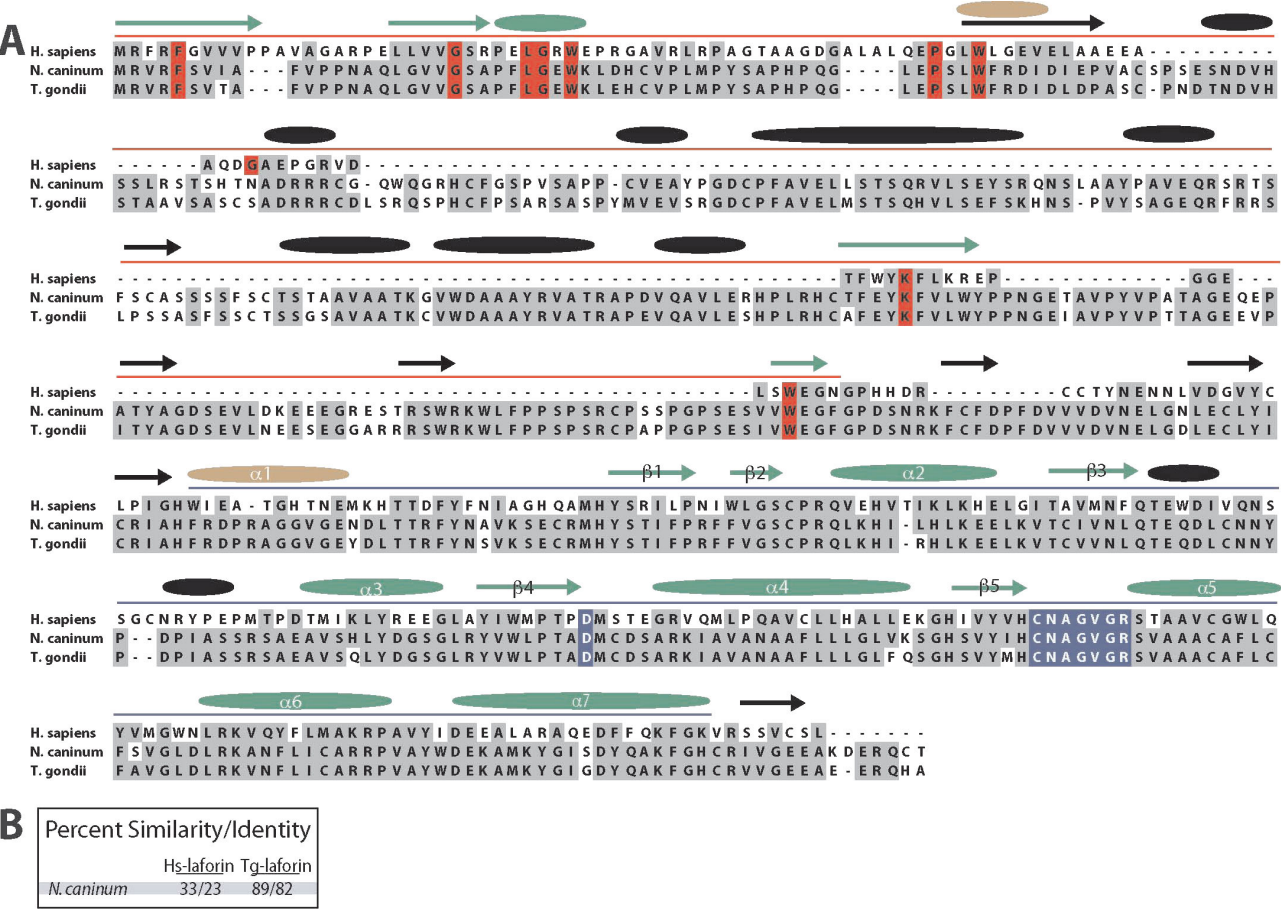
**Conservation of laforin in *N. caninum***. *A*, An alignment of *N. caninum *(Nc-) laforin, with *T. gondii *(Tg-) laforin, and *H. sapiens *(Hs-) laforin. Residues boxed in red are invariant CBM20 residues as defined by the CBM20 family [13], and residues boxed in blue are part of the DSP catalytic site. Residues boxed in dark gray are identical. Accession numbers are listed in Additional File 8. The red line above the sequence delineates the CBM and the blue line delineates the DSP. The predicted secondary structure is represented by arrows (β-sheets) and ovals (α-helices). Green arrows and ovals represent a match with the predicted secondary structure of Hs-laforin, black arrows and ovals are shared between Nc-laforin and Tg-laforin, and brown are unique for Hs-laforin. The β-sheets and α-helices of the DSP are numbered according to standard DSP nomenclature [11]. *B*, Percent similarity and identity of Nc-laforin with Hs- and Tg-laforin.

The only novel putative laforin ortholog that we uncovered was in the genome of *Neospora caninum*, a genome we predicted would contain laforin [15] (Fig. 2A). *N. caninum *laforin (Nc-laforin) was only16% and 23% similar to Cm- and Hs-laforin, respectively (Fig. 2B). As we predicted, *N. caninum *fulfils our three criteria in that it is of red algal descent, possesses true mitochondria, and generates insoluble starch in the form of amylopectin granules [32-35]. *N. caninum *is classified as an alveolate and is a parasite of canines [36]. *N. caninum *structurally resembles *T. gondii *to such a degree that it was not properly identified until 1988 [37]. Therefore, we aligned the putative Nc-laforin with *T. gondii *(Tg)-laforin and found that these proteins are 82% identical (Fig. 2). In addition, all of the residues known to be necessary for glucan binding and phosphatase activity in Tg-laforin are conserved in Nc-laforin (Fig. 2A). Furthermore, the predicted secondary structure of Nc-laforin and Tg-laforin are identical and both are very similar to Hs-laforin (Fig. 2A). Given the degree of similarity between Nc- and Tg-laforin at the primary and secondary amino acid level and the fact that we previously cloned and characterized Tg-laforin [15], Nc-laforin is a laforin ortholog. This finding confirms the predictive power of our three criteria in predicting the absence or presence of laforin in protozoan genomes. We did not uncover a laforin ortholog in the genomes of *G. sulphuraria*, *G. theta*, or *S. neurona*, but their nuclear genomes are not complete and we still predict that they do contain laforin.

### Discovery of putative laforin orthologs in invertebrate genomes

Metazoans are defined as all living animals that contain tissues and are descended from the last common ancestor of Bilateria, Cnidaria (jellyfish, sea anemones, corals, hydra, etc.), Ctenophora (comb jellies), Placozoa (*Trichoplax *sp.), and Porifera (sponges) [38]. Bilateralians are subdivided as either protostomes (including arthropods, nematodes, annelids, and mollusks) or deuterostomes. The three major deuterstome phyla, chordates, echinoderms (sea urchins, sea stars, etc.), and hemichordates (acorn worm), arose from a common ancestor more than 600 million years ago, followed by subsequent divergence of the chordates into three subphyla: cephalochordates, urochordates (also called tunicates), and vertebrates (summarized in Additional File 2) [39,40].

The gene encoding laforin is an evolutionarily ancient gene, originating in a primitive red alga, or its ancestor, long before the emergence of metazoans [15]. While laforin is an ancient gene, it has a unique evolutionary lineage. Although we previously identified laforin orthologs in five protozoan genomes, we did not find it in any non-vertebrate model organism genomes (yeast, fly, or worms), nor did we find it in the genome of any invertebrates [15]. We postulated that invertebrates lack laforin because they do not synthesize an insoluble glucan as an energy source (as do the protozoans that contain laforin) and they do not inhibit insoluble glucan accumulation (as seen with vertebrates inhibiting LBs). However, over the course of the last two years multiple basal position metazoan genomes have been sequenced or improved and a surplus of information has been gleaned by the evo-devo community from the genomes of *Branchiostoma floridae *(commonly known as amphioxus, subphylum cephalochordata), *Ciona intestinalis *(sea squirt belonging to urochordates), *Monosiga brevicollis *(choanoflagellate and closest known unicellular relative to metazoans), *Nematostella vectensis *(sea anemone belonging to the ancient metazoan Phylum Cnidaria), and *Trichoplax adhaerens *(arguably the simplest free-living metazoan, Phylum Placozoa) [41-45]. While definitive conclusions concerning the origin and early radiation of these organisms in the metazoan tree of life remain unsettled, these reports have elucidated multiple aspects of metazoan evolution and the genomes of primitive metazoans regarding genome complexity, exon-intron structure, gene repertoire, and rates of molecular evolution.

**Figure 3:**
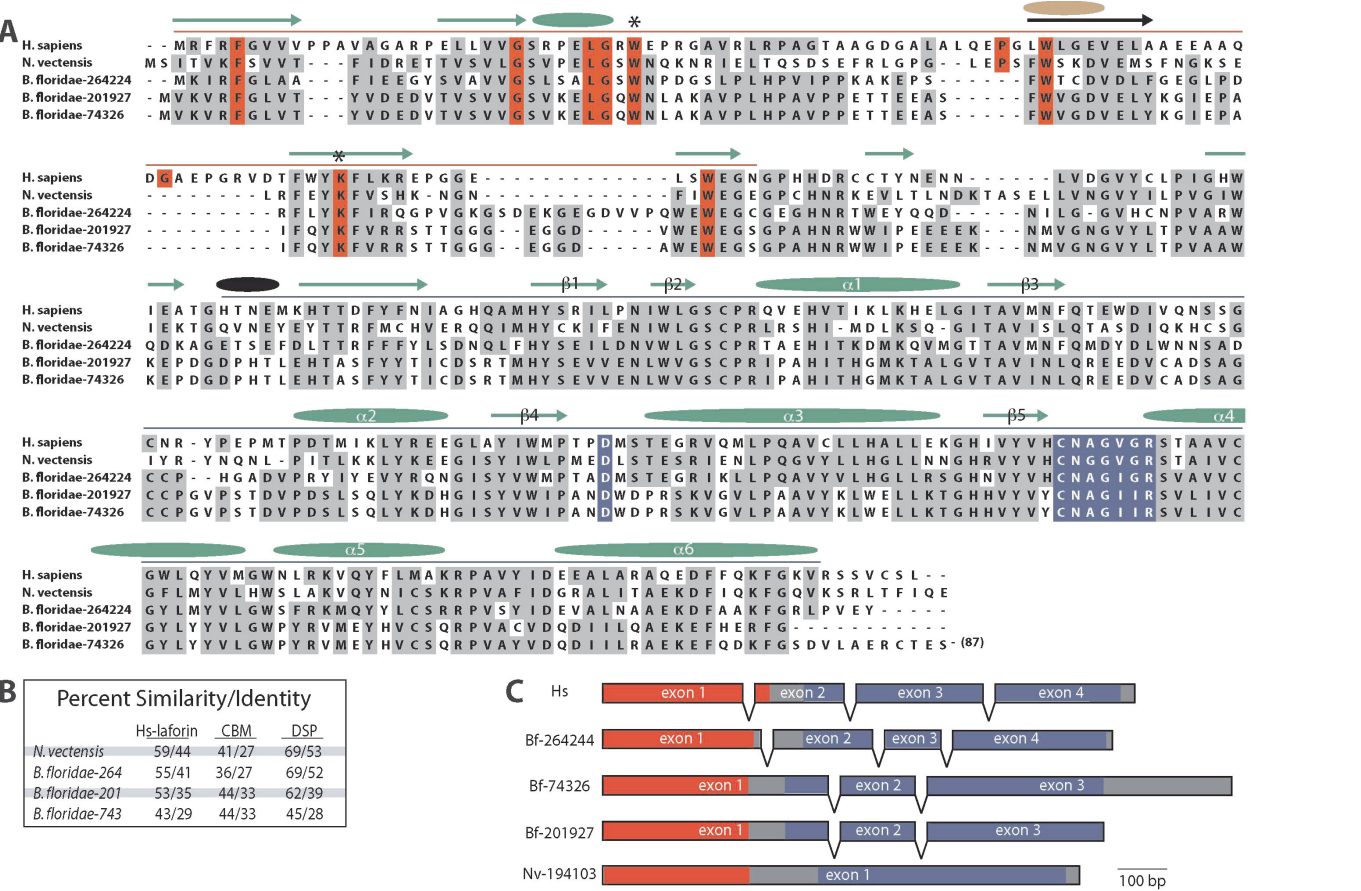
**Laforin orthologs in invertebrates**. *A*, An alignment of *N. vectensis *(Nc-) laforin and three *B. floridae *(Bf-) laforin with *H. sapiens *(Hs-) laforin. Highlighted residues and shapes representing predicted secondary structure are as in Fig. 2. The red line above the sequence delineates the CBM and the blue line delineates the DSP. Green arrows and ovals represent a match with the predicted secondary structure of Hs-laforin, black arrows and ovals are shared between Nv-laforin and Bf-laforin, and brown are unique for Hs-laforin. The β-sheets and α-helices of the DSP are numbered according to standard DSP nomenclature [11]. The two asterisks indicated residues necessary for carbohydrate binding. *B*, Percent similarity and identity of Bf- and Nv-laforin with Hs-laforin. *C*, Predicted intron-exon boundaries for the genes encoding Hs-, Bf-, and Nv-laforin. The coding region that encodes the CBM is highlighted in red, the DSP in blue, and the rest is in gray.

Given these recent advances we performed searches as described above searching multiple databases (Additional File 1) for putative laforin orthologs in the genome of 212 eukaryotes (including the 122 protozoan). We identified putative laforin orthologs in the genome of two invertebrates, the cnidarian sea anemone *Nematostella vectensis *and the small worm-like urochordate *Branchiostoma floridae *(Fig. 3A). The putative *N. vectensis *laforin (Nv-laforin) is 44% identical to Hs-laforin and contains all but one of the known residues conferring glucan binding and phosphatase activity (Fig. 3A &3B). In the *B. floridae *genome we discovered three open reading frames that each encoded a putative laforin ortholog (Fig. 3A). The putative *B. floridae *laforin (Bf-laforin) orthologs are 29–41% identical to Hs-laforin and share the majority of residues important for glucan binding and phosphatase activity (Fig. 3A &3B). In addition, all four genes encode proteins that have very similar predicted secondary structure to Hs-laforin (Fig. 3A). The predicted secondary structure of the CBM of Nv-laforin and the Bf-laforins are nearly identical with Hs-laforin, but have a predicted helix in place of a coil-coil (Fig. 3A). All four proteins contain the predicted five β-sheets and six α-helices typical of a dual specificity phosphatase domain and are identical with Hs-laforin (Fig. 3A).

To gain insight into the evolution of these putative laforin orthologs, we analyzed the gene structure and intron-exon boundaries of each and compared them with the gene encoding Hs-laforin, *EPM2A*. *EPM2A *is comprised of four exons and three introns (Fig. 3C) [9,10]. Similarly, Bf-laforin-264244 has the same arrangement and the intron-exon boundaries are at similar locations (Fig. 3C). The other two putative Bf-laforin orthologs each contain three exons and two introns. Additionally, exon 1 from these two Bf-laforins is similar in size to exon 1 and exon 2 of both Hs-laforin and Bf-laforin-264244, suggesting possible intron loss (Fig. 3C). Finally, Nv-laforin has no introns, suggesting intron loss and potentially an increased rate of evolution at this locus in *Nematostella*.

Accelerated rates of evolution occur in echinoderms, fruit flies, nematodes, and the sea squirt *C. intestinalis *[40,46-48]. These accelerated rates of evolution result in amino acid substitution, intron loss, gene loss, and genome rearrangement. Surprisingly, recent studies show that the genomes of *Nematostella *and *Branchiostoma *have evolved at a rate comparable to or slower than vertebrates [42-44]. These studies generated phylogenies derived from 104 [43], 337 [44], and 1090 [42] single-copy nuclear encoded genes to estimate rates of molecular evolution. Their phylogenies showed long branch lengths, indicating increased sequence divergence, for the genomes of all organisms investigated except vertebrates, *Nematostella*, and *Branchiostoma *[42-44]. Thus, they concluded that the genomes of the fly, hydra, nematode, yeast, sea squirt, sponge, snail, and the choanoflagellate *M. brevicollis *have evolved at a more rapid rate (summarized in Table 1). In addition to primary sequence comparison, these studies also examined the conservation of introns in various species and found that in alignable regions the genomes of *Nematostella *and *Branchiostoma *share >80% of human introns [42,44]. Conversely, *D. melanogaster*, *C. elegans*, and *C. intestinalis *have lost 50–90% of the inferred ancestral metazoan introns [42,44] (summarized in Table 1). Thus, it is proposed that ancient metazoan genomes more closely resembled the complexity seen in the human genome rather than that observed in yeast, flies, or worms [42-45,49].

**Table 1:** Molecular evolution and conservation of lafoin.

**Organism**	**laforin gene**	**moleuclar evolution**	**reference**
*Branchiostoma*	**present**	slow	42
*Ciona intestinalis*	absent	accelerated	42, 44, 48
*Caenorhabditis elegans*	absent	accelerated	44, 46, 48
*Drosophila*	absent	accelerated	40, 43, 44, 46, 48
Echinoderms	absent	accelerated	40
*Hydra*	absent	accelerated	43, 44
*Monosiga brevicollis*	absent	accelerated	43, 44
*Nematostella*/Cnidaria	**present**	slow	42, 43, 44, 46, 47
sponge	absent	accelerated	43, 44
vertebrates	**present**	slow	40, 42, 43, 44, 46, 47, 48
yeasts	absent	accelerated	44

Rates of molecular evolution are defined in these studies as either substitutions per amino acid and/or position and phase of introns

The laforin gene in *H. sapiens*, *T. gondii*, *N. caninum*, *E. tenella*, *P. tetraurelia*, and *B. floridae *all contain at least four exons. Therefore, we propose that the laforin gene originally had four or more exons, and that the introns were lost in *C. merolae *and *Nematostella*. These results suggest that the laforin gene locus underwent a higher rate of molecular evolution than genes in *Nematostella *that share conserved introns with human genes. This increased molecular evolution may explain the absence of laforin in yeast, flies, worms, and the majority of invertebrates as we discuss below.

### Biochemical characterization of Nv- and Bf-laforin

To determine if we had identified true laforin orthologs in invertebrates, we cloned the genes expressing Nv-laforin and Bf-laforin-264244 from *Nematostella *and *Branchiostoma*, respectively. We previously cloned and characterized Tg-laforin and found that we could only obtain soluble recombinant Tg-laforin when we added a GST tag to the amino terminus of Tg-laforin [[20], and unpublished data]. Even with the addition of the GST tag, the majority of GST-Tg-laforin is insoluble, suggesting that the majority of the protein does not correctly fold in bacteria. Unlike Hs-laforin but similar to Tg-laforin, both Nv- and Bf-laforin were largely insoluble with a HIS6 epitope. Therefore, we generated bacterial constructs expressing GST-Nv-laforin-HIS_6 _and GST-Bf-laforin-HIS6, purified the recombinant proteins (Additional File 3), and biochemically characterized them.

**Figure 4:**
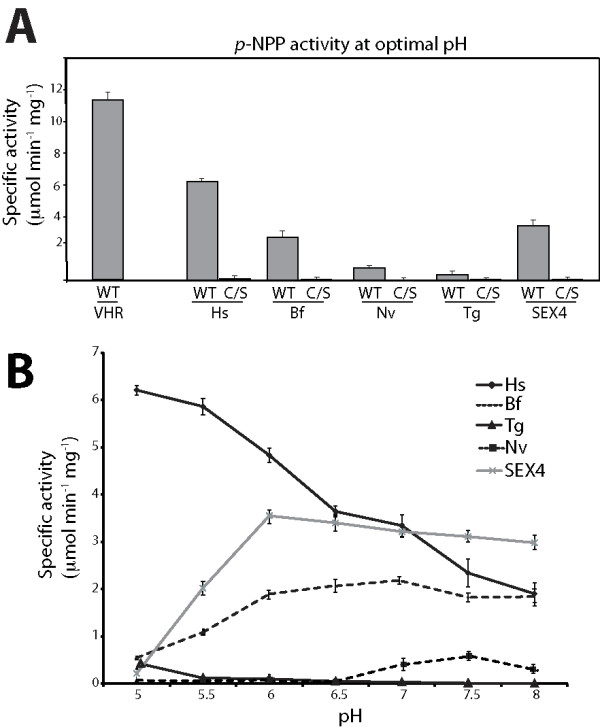
**Invertebrate laforin orthologs possess *p*-NPP activity**. *A*, Specific activity of VHR, Hs-laforin, Hs-laforin-C/S, Bf-laforin, Bf-laforin-C/S, Nv-laforin, and Nv-laforin-C/S at their respective optimal pH. WT, wild type. *B*, Specific activity of VHR, Hs-laforin, Bf-laforin, and Nv-laforin at pH units 5–8. Error bars indicate mean ± SD.

Characteristic of all dual specificity phosphatases, human DSP VH1-related (VHR) and Hs-laforin exhibit phosphatase activity against the artificial substrate para-nitrophenylphosphate (p-NPP; Fig. 4A) [12,50]. Both Bf- and Nv-laforin also utilized p-NPP as an artificial substrate (Fig. 4A). Bf-laforin exhibited a similar specific activity as Hs-laforin and SEX4, while the activity of Nv-laforin was lower and similar to Tg-laforin. The decreased activity of Nv-laforin is likely due to our difficulty in generating soluble Nv-laforin and suggests that some of the soluble Nv-laforin may be misfolded, similar to Tg-laforin. Even though the activity of Nv-laforin is substantially decreased, it does exhibit phosphatase activity and therefore it is a phosphatase. DSPs act by forming a phosphoenzyme intermediate after nuleophilic attack of the phosphorus atom of the substrate by a cysteine residue that is conserved in all DSPs in their signature motif, H**C**xxGxxRS (CX5R) [51]. Mutation of this cysteine renders DSPs catalytically inactive. Therefore, we generated cys/ser (C/S) mutations in Bf- and Nv-laforin to test if their activity was dependent on this cysteine. As predicted, the Bf- and Nv-laforin C/S mutants displayed no activity against p-NPP (Fig. 4A). Enzymes generally have a preferred pH within to act, and DSPs are no different. We tested the phosphatase activity of Bf- and Nv-laforin at 0.5 unit increments from pH 5–8. While Hs-laforin has a maximal activity against p-NPP at pH 5, Bf-laforin had a maximal activity at pH 7 and Nv-laforin at pH 7.5 (Fig. 4B).

**Figure 5:**
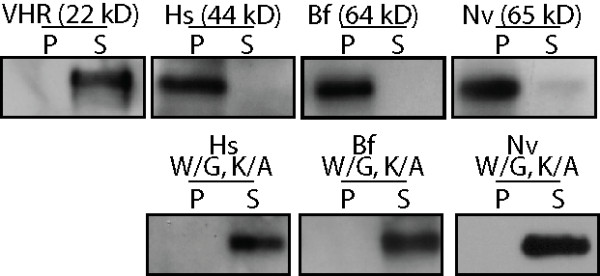
**Invertebrate laforin orthologs bind amylopectin**. Recombinant proteins were incubated with 5 mg/ml amylopectin, amylopectin was pelleted by ultracentrifugation, and proteins in the pellet (P) and supernatant (S) were visualized by Western analysis. VHR, *H. sapiens *VHR; Hs, Hs-laforin; Bf, Bf-laforin; Nv, Nv-laforin. Mutated residues are marked with an asterisk in Fig. 3.

Since laforin is the only phosphatase in any Kingdom Animalia genome with a CBM, it is predicted to be the only phosphatase that binds carbohydrates. Bf- and Nv-laforin bound amylopectin to the same extent as Hs-laforin (Fig. 5). Conversely, VHR, which lacks a CBM, did not bind amylopectin (Fig. 5). Dixon and colleagues previously demonstrated that conserved tryptophan and lysine residues (marked with asterisks in Fig. 3) that participate in binding to the sugar are necessary for Hs-laforin to bind amylopectin (Fig. 5) [13]. Accordingly, mutation of these corresponding residues in Bf-laforin (W29 and K69) and Nv-laforin (W31 and K76) also abolished their ability to bind amylopectin (Fig. 5).

**Figure 6:**
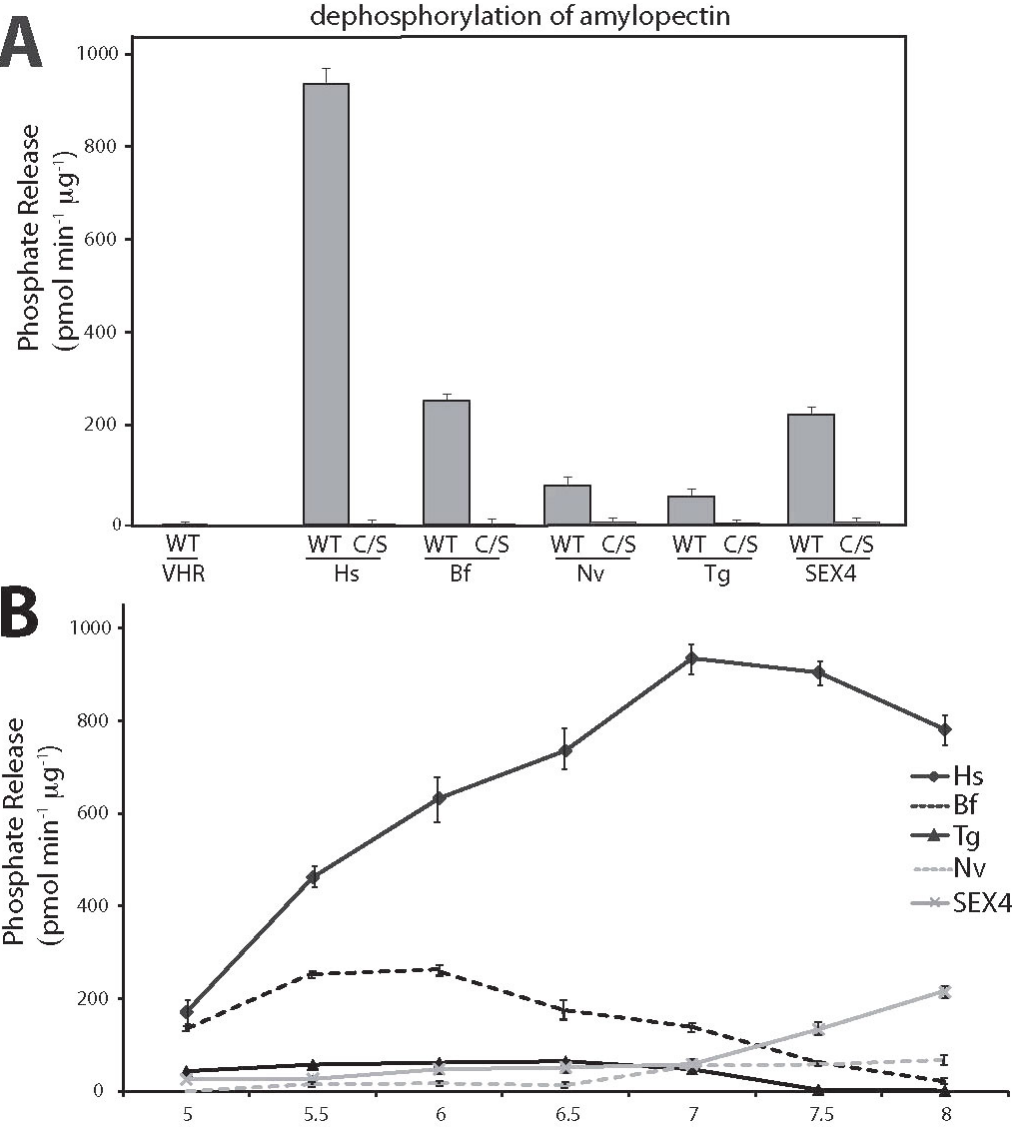
**Invertebrate laforin orthologs release phosphate from amylopectin**. *A*, Phosphate release measured by malachite green assays using VHR, Hs-laforin, Hs-laforin-C/S, Bf-laforin, Bf-laforin-C/S, Nv-laforin, and Nv-laforin-C/S against amylopectin at their respective optimal pH. WT, wild type. *B*, Specific activity of VHR, Hs-laforin, Bf-laforin, and Nv-laforin at pH units 5–8. Error bars indicate mean ± SD.

Some DSPs dephosphorylate non-proteinaceous substrates, such as phosphatase and tensin homologue (PTEN), the myotubularin family, and Sac domain phosphatases that all dephosphorylate the inositol head group of phospholipids [52-56]. We recently discovered that laforin belongs to a unique class of enzymes that dephosphorylates glucans, namely glycogen and starch, and that other classes of phosphatases lack this activity [15,21]. Amylopectin is the major component of plant starch and contains detectable amounts of phosphate [57,58]. Therefore, we tested if Bf- and Nv-laforin could liberate phosphate from amylopectin. Similar to SEX4 and Hs-laforin, Bf- and Nv-laforin liberated phosphate from amylopectin, whereas VHR did not hydrolyze phosphate from amylopectin (Fig. 6A). The activity of Bf-laforin was comparable to the activity of SEX4, and the activity of Nv- and Tg-laforin were comparable. Similar to the p-NPP data, the activity of Bf- and Nv-laforin against amylopectin was also pH dependent (Fig. 6B).

Bf- and Nv-laforin possess the same three *in vitro *biochemical properties as Hs-laforin: both utilize p-NPP as an artificial substrate, bind amylopectin, and liberate phosphate from amylopectin. Additionally, the invertebrate laforin orthologs contain the critical signature primary amino acids of both a CBM20 and DSP, and they possess the same predicted secondary structure as Hs-laforin. Thus, our bioinformatics searches for a protein containing a CBM and DSP correctly predicted the proteins biochemical properties and we identified novel laforin orthologs. Our finding of laforin orthologs in two invertebrate genomes reinforces the global function of this protein and yields insights into the evolution of this gene family.

### Evolutionary lineage of glucan phosphatases

As discussed above, we previously reported that laforin is not confined to vertebrate genomes, but it is also conserved in five protozoan genomes [15]. The gene encoding laforin is conserved in species as divergent as humans and red algae, but it is absent in the vast majority of protozoan and invertebrate genomes. *SEX4 *is conserved in diverse members of Archaeplastida/Kingdom Plantae and is necessary for proper starch metabolism in *Arabidopsis *[15,19,20]. These findings suggest that laforin and SEX4 are ancient proteins that regulate an aspect of energy metabolism conserved in multiple kingdoms, namely the dephosphorylation of glycogen and starch.

We previously postulated that throughout evolution organisms maintained or lost laforin depending on their "need" to manage insoluble glucans [15]. We suggested that protists with laforin maintained it to manage insoluble glucans as an energy source [15]. Similarly, vertebrates maintained laforin because of their "need" to combat Lafora body accumulation and used this reasoning to explain why the genome of most metazoans (i.e. animals) lack laforin [20]. However, it is also possible that vertebrate genomes have laforin as a result of horizontal gene transfer (HGT) from a protist.

**Figure 7:**
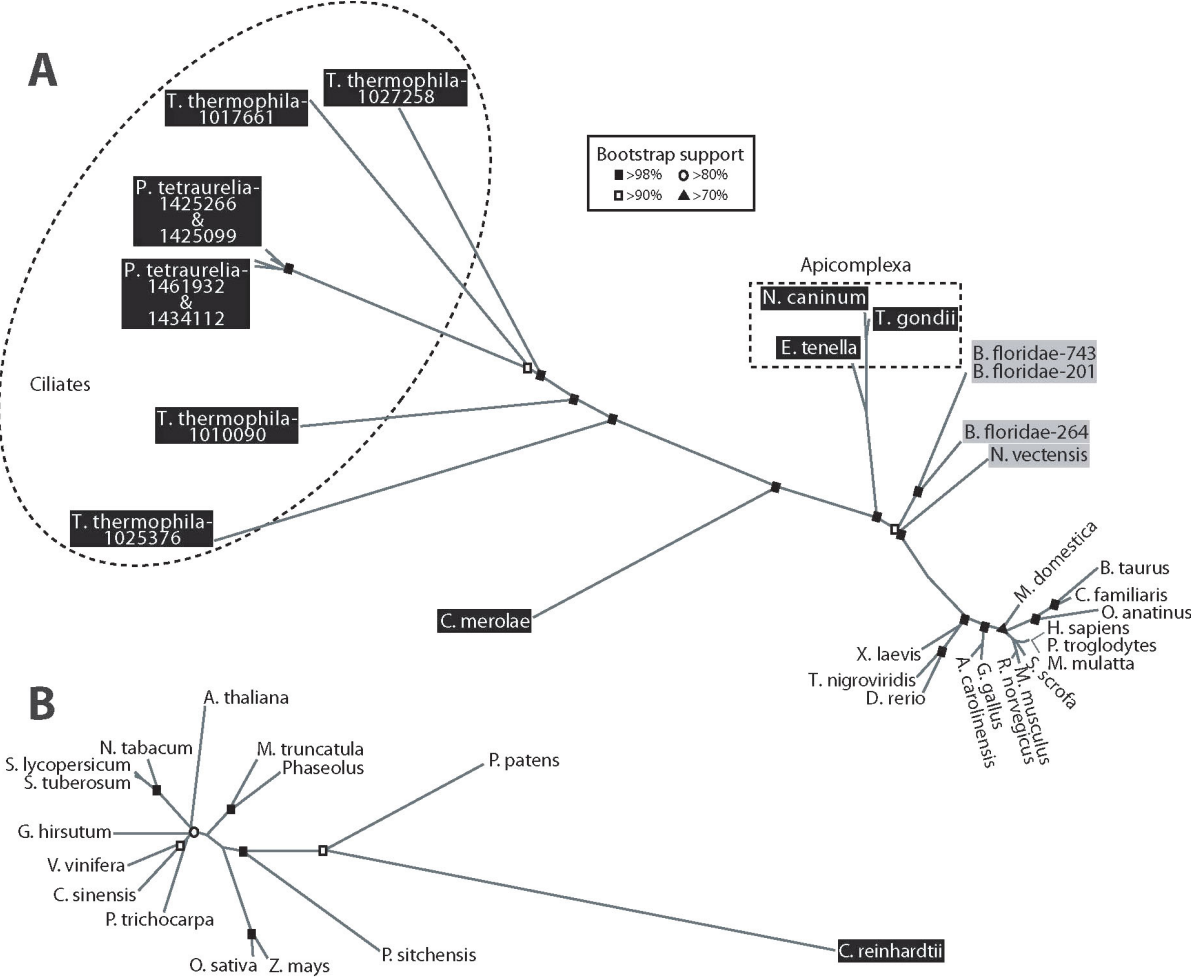
**Phylogeny of laforin laforin orthologs**. *A*, Unrooted phylogeny of all laforin orthologs to date was generated as described in Methods, and accession numbers are listed in Additional File 8. Organisms boxed in black are protists, organisms boxed in gray are invertebrates, and those with no box are vertebrates. Bootstrap values are listed at major nodes as indicated in the inset. *B*, Unrooted phylogeny of all SEX4 orthologs to date was generated as in *A*, and accession numbers are listed in Additional File 6. Organisms boxed in black are single-cell, green algae. Bootstrap values are as in *A*.

To further investigate the evolutionary heritage of laforin and SEX4 we generated a phylogeny of all laforin and SEX4 orthologs. We identified full-length laforin orthologs in fifteen vertebrate genomes (encompassing all five classes), two invertebrate genomes, and six protozoan genomes (Fig. 7A). Additionally, we found multiple laforin paralogs in the genomes of *B. floridae*, *T. thermophila*, and *P. tetraurelia*. The laforin paralogs have an array of intron-exon numbers and boundaries, from a single exon to similar numbers (4 exons) and boundaries as Hs-laforin to as many as eight exons (Fig. 3C & Additional File 4). Gene duplication and intron gain and loss are all indicative of increased molecular evolution, suggesting that the laforin locus in these organisms has experienced increased evolutionary pressures. Among the protists, the Apicomplexa laforin orthologs rooted much closer to the invertebrates and vertebrates than did the Ciliate laforin orthologs (Fig. 7A). The Apicomplexa genomes all contain a single ortholog, while the two Ciliates both contain three. Both of these results argue for an increased rate of evolutionary drift in the Ciliates at the laforin locus. The alveolates that have laforin (see Fig. 1C) all contain at least four exons and three introns, thus agreeing with reports from the Koonin laboratory demonstrating increased intron-rich genes in alveolates [59].

In searching for SEX4 orthologs, we identified full-length orthologs in fourteen genomes, encompassing trees, land plants, a moss (*Physcomitrella patens*), and a single-cell green alga (Fig. 7B). In addition, we identified several partial cDNA or protein hits of SEX4 orthologs in other Archaeplastida (data not shown). These results demonstrate that *SEX4 *is conserved in all branches of Archaeplastida/Kingdom Plantae and highlight the importance of this glucan phosphatase throughout Archaeplastida.

Our finding of laforin in two invertebrate genomes raises questions regarding the evolutionary lineage of laforin in metazoans/animals. It is possible that the unique evolutionary lineage of laforin was a result of HGT from a protozoan or its ancestor. If so, this event occurred earlier than the radiation of metazoans because laforin is conserved in two invertebrate genomes, including the Cnidarian *Nematostella*.

An alternative hypothesis, and one that we favour, postulates that in non-protozoan genomes the absence or presence of laforin was determined by rates of molecular evolution and the need to combat insoluble glucans, while in protists conservation of laforin is determined by the three criteria previously presented and discussed [15]. This hypothesis seems likely given recent reports showing the rate of molecular evolution in *Branchiostoma *and *Nematostella *is similar to that of vertebrates and the rate is much higher in other invertebrates, as discussed above (Table 1).

This hypothesis calls into question why laforin has been conserved. Invertebrates may not need laforin because they have significantly shorter lifespans than vertebrates. In support of this thought, the only cellular pathology observed in LD patients is neuronal apoptosis [3]. Since neurons live longer than most all other cell types, it seems plausible that invertebrates may be more like skin cells (or other cell types with shorter lifespans); they accumulate LBs but not to a detrimental state. Thus, laforin may represent a vestigial gene or perhaps a pseudogene in *Nematostella *and *Branchiostoma*.

Alternatively, *Nematostella *and *Branchiostoma *may have laforin for a purpose. Vertebrates do not utilize insoluble glucans as an energy source, but they do "combat" the accumulation of insoluble glucans, in the form of detrimental LBs. We know that many, if not all, vertebrate species suffer from LD (including, canines, felines, cattle, and birds) [60-64]. It is possible that *Nematostella *and *Branchiostoma *also have laforin in order to combat LBs.

Lastly, *Nematostella *and *Branchiostoma *may have laforin because their genomes have not evolved as rapidly as other invertebrates, as has been recently demonstrated, and thus still have/need laforin [42,44]. If this is the case, other invertebrates (e.g flies and worms) may not need laforin because they have evolved a separate means to deal with LB-like accumulations.

## Conclusion

Regardless of what has driven the evolutionary lineage of laforin, these results define the boundaries of glucan phosphatase conservation. These findings coupled with our previous work establish the conservation of laforin and SEX4 in evolutionary niches from protozoans to plants to algae to vertebrates and now invertebrates. Notably, laforin and SEX4 are absent in bacteria and archaea. While these groups do contain each domain that is found in glucan phosphatases (i.e. a phosphatase domain and carbohydrate binding domain), no protein in their genomes contains both. Given the completeness of bacterial genomes, it is unlikely that a glucan phosphatase exists in bacteria. Therefore, we suspect that we have identified the complete evolutionary lineage of the glucan phosphatases laforin and SEX4. The conservation of laforin across evolutionary niches, coupled with what appears to be complete conservation of SEX4 throughout all Archaeplastida/Kingdom Plantae demonstrate that phosphorylation/dephosphorylation of glucans is pervasive throughout nature.

## Methods

### Plasmids and Proteins

Wild type and C/S Hs-laforin in pET21a (Novagen, San Diego, CA) for use in bacterial expression were described previously [13,65]. Recombinant HIS-tagged VHR cloning and purification have been previously described [21]. The complete open reading frame of Bf-laforin-264224 was amplified from cDNA provided by the *Branchiostoma floridea *Gene Collection [66]. The complete open reading frame of Nv-laforin was amplified from *Nematostella *DNA provided by Dr. Mark Q. Martindale. Bf- and Nv-laforin were cloned into pET-GST_X _and pET21a, respectively [67]. Mutations were introduced using QuickChange (Stratagene). Recombinant GST- and His-tagged proteins were expressed in Escherichia coli BL21 (DE3) CodonPlus RIL cells (Stratagene, La Jolla, CA) and purified using Ni^2+^-agarose (Qiagen, Germany) and/or glutathione-agarose affinity chromatography steps as described previously [68].

### Phosphatase Activity Assays

Hydrolysis of *para*-nitrophenylphosphate (*p*-NPP) was performed in 50 μl reactions containing 1X phosphate buffer (0.1 M sodium acetate, 0.05 M bis-Tris, 0.05 M Tris-HCl, 2 mM dithiothreitol, at the appropriate pH), 50 mM *p*NPP, and 100–500 ng of enzyme at 37°C for 5–30 minutes. The reaction was terminated by the addition of 200 μl of 0.25 M NaOH and absorbance was measured at 410 nm. We tested the specific activity of each enzyme at pH 5.0, 5.5, 6.0, 6.5, 7.0, 7.5, and 8.0. Malachite green assays were performed as described [69] with the following modifications: 1X phosphate buffer, 100–500 ng of enzyme, and ≈45 μg of amylopectin in a final volume of 20 μl. The reaction was stopped by the addition of 20 μl of 0.1 M *N*-ethylmaleimide and 80 μl of malachite green reagent. Absorbance was measured after 30 minutes at 620 nm. We tested the specific activity of each enzyme at the same pH units as above.

### Carbohydrate binding Assay

Carbohydrate binding assays were done similarly as described previously [13]. Briefly, 50 mg of amylopectin was dissolved in 400 μl of ethanol, followed by the addition of 1 ml of water and 1 ml of 2 M NaOH, 2 ml of water was added, pH was adjusted to 6.5, and the total volume was brought to 10 ml. 1 ml of 5 mg/ml amylopectin was centrifuged at 50 K for 1.5 hours, the pellet was resuspended in 0.5 ml of buffer (50 mM Tris, pH7.5, 150 mM NaCl, 0.1% β-mercaptoethanol) with protease inhibitors, 0.5 μg of recombinant protein was added, the tube was rotated at 4°C for 1 hour, a second centrifugation was preformed at 50 K for 1.5 hours, the proteins in the supernatant were precipitated with acetone, and the pellet was resuspended in Western loading dye. Potato amylopectin was purchased from Sigma (St. Louis, MO). Recombinant proteins were detected with α-HIS-horeseradish peroxidase (HRP) antibody (Santa Cruz) and SuperSignal West Pico (Pierce).

### Search strategy, sequence alignment, and phylogenetic analyses

The sequences of laforin and SEX4 orthologs were obtained by performing tBLASTn searches using the GenBank "dbEST" database or BLASTp and PSI-BLAST [70] searches using GenBank "eukaryote genome" and "non-redundant" (nr) databases, the *Cyanidioschyzon merolae *genome project, Department of Energy Joint Genome Institute Resource, The Institute for Genomic Research (TIGR), ToxoDB, GeneDB, Genoscope, UCSC Genome Browser, *Tetrahymena *Genome Database, and multiple organism specific databases. Accession numbers for small subunit (SSU) ribosomal RNA (rRNA), SEX4 orthologs, and laforin orthologs are listed in Additional File 5, 6, and 8, respectively. The web address for each database is listed in Additional File 4. A list of each genome that we investigated and a reason why an organism's genome lacks laforin is listed in Additional File 7. Amino-acid sequences of laforin orthologs were aligned by ClustalW [71] and refined manually using MacVector. SSU rRNA sequences were obtained by performing BLASTn using GenBank from "all organisms" and "nr" databases and accession numbers are listed in Additional File 5. Phylogenetic trees were generated from a ClustalW [71] multiple sequence alignment using PROTDIST and FITCH from the PHYLIP 3.65 software package and displayed utilizing HYPERTREE 1.0.0 [72].

## Authors' contributions

MSG initiated the study, cloned the various laforin orthologs, designed the experiments, performed experiments, performed all evolutionary analyses, compiled and analyzed data, generated figures, directed the project, and wrote the manuscript. RMP subcloned laforin orthologs into vectors, performed *in vitro *biochemical assays, assisted with interpretation of results, and assisted with writing that paper and generating figures. Both authors read and approved the final manuscript.

## Supplementary Material

Additional file 1 **Table of Non-NCBI databases used in this study**. The non-NCBI databases used to search for laforin orthologs are listed in blue and the organisms' genome in each database is listed in black. Many of the databases were found by performing a Google search of the organisms genus name and "genome." Click here for file

Additional file 2 **Summary of metazoan phylogeny**. This is a depiction of an unrooted phylogeny showing the major classifications of metazoans (note: not to scale). Highlighted in yellow are organisms or groups of organisms that contain laforin. *Monosiga brevicollis *is not a metazoan, but is arguably the closest unicellular relative to metazoans. For a broad frame of reference visit . Click here for file

Additional file 3 **Purification of recombinant GST-Bf-laforin and GST-Nv-laforin**. *A*, GST-Nv-laforin-HIS_6 _was purified from soluble *E. coli *lysate via Ni^2+^-agarose affinity chromatography. *B*, GST-Bf-laforin-HIS_6 _was purified from soluble *E. coli *lysate via Ni^2+^-agarose affinity chromatography. U, uninduced; I, induced; PL, post-lysis; P, pellet (insoluble) fraction; S, soluble fraction; NB, not bound to beads; Bds, bound to HIS beads; HIS E, HIS eluate; GST E, GST eluate. Click here for file

Additional file 4 **Intron-exon numbers and boundaries of laforin genes**. Predicted intron-exon boundaries for the genes encoding Hs-, Tg-, Nc-, Et-, Cm-, Tt- and Pt-laforin. The coding region that encodes the CBM is highlighted in red, the DSP in blue, and the rest is in gray. Accession numbers are listed in Additional File 8. Click here for file

Additional file 5 **Table of small subunit (SSU) ribosomal RNA (rRNA) accession numbers**. Listed on the left are the organisms from the phylogeny in Figure 1 and on the right are the accession numbers for the SSU rRNA genes. Each of the accession numbers are from NCBI Genbank unless otherwise noted. Click here for file

Additional file 6 **Table of accession numbers for SEX4 orthologs.** Click here for file

Additional file 7 **Table of genomes investigated for the presence of laforin**. The genome of each organism was searched for laforin and/or SEX4 using the appropriate database (Additional File 4). If laforin was absent an extensive literature search was performed on the organism to determine which of the three criteria it lacked: red algal descent, mitochondrion, and/or floridean starch/LBs. If the organism lacked laforin, then at least one of the three criteria that it lacks is presented beside its name. The organism name of genomes containing laforin are bold and in green. The organism name of genomes that are nearing completion and that contain laforin based on our predictions are bold and in red. The phrase "incomplete genome, has laforin" refers to organisms with incomplete genomes, but where a partial CBM and DSP corresponding to laforin was found. Highlighted in yellow are organisms found in this study to contain laforin. Organism classification is based on widely accepted taxonomy classifications [73]. Click here for file

Additional file 8 Table of accession numbers for laforin orthologs. Click here for file
