# Supplementary material for: Conservation of the glucan phosphatase laforin is linked to rates of molecular evolution and the glucan metabolism of the organism
Source: BMC Evol Biol. 2009 Jun 22;9:138. doi: 10.1186/1471-2148-9-138 (PMC2714694; doi:10.1186/1471-2148-9-138)
Supplement: Additional file 1 — Table of Non-NCBI databases used in this study. The non-NCBI databases used to search for laforin orthologs are listed in blue and the organisms' genome in each database is listed in black. Many of the databases were found by performing a Google search of the organisms genus name and "genome." [file 1471-2148-9-138-S1.pdf]

Additional File 4 Non-NCBI databases.

Kingdom Animalia databases

D. discoideum, *E. histolytica*, *E. tenella*, *Leishmania sp.*, *N. caninum, Plasmodium* sp., *T. annulata, Trypanosome* sp., *S. mansoni*:

http://www.genedb.org/

B. floridae, C. intestinalis, Capitella sp., D. pulex, *E. huxleyi*, *H. robusta*, *L. bicolor, L. gigantea, M. brevicollis, M. graminicola, N. gruberi, N. haematococca, N. vectensis, O. tauri, P. stipitis, P. tricornutum, T. pseudonana, Trichoplax sp., V. carteri:*

http://genome.jgi-psf.org/

B. bovis, B. malayi, C. parvum, D. discoideum, E. histolytica, E. tenella, F. hepatica, H. contortus, L. seymouri, Leishmania sp., Plasmodium sp., S. neurona., S. mansoni, T. vaginalis, Trypanosoma sp., T. gondii:

http://tigrblast.tigr.org/tgi/

Ascaris, B. malayi, B. bigemina, *C. deanei*, *D. discoideum*, *Entamoeba sp*, *E. tenella*, *H. contortus, Leishmania sp.*, *N. caninum, Plasmodium* sp., *Schistosoma sp., S. scrofa, Strongyloides sp., Trypanosome* sp., *T. annulata, T. parva*:

http://www.sanger.ac.uk/DataSearch/blast.shtml

A. carolinensis, B. floridae, C. intestinalis, M. domestica, O. anatinus, P. marinus:

http://genome.ucsc.edu/cgi-bin/hgBlat

A. gambiae:

http://www.genoscope.cns.fr/externe/English/Projets/Projet_AK/organisme_AK.html

C. intestinalis	*http://ghost.zool.kyoto-u.ac.jp/indexr1.html*
C. merolae:	http://merolae.biol.s.u-tokyo.ac.jp/
C. parvum:	http://cryptodb.org/cryptodb/
D. discoideum:	*http://dictybase.org/*
Diatom ESTs:	http://avesthagen.sznbowler.com/
E. tenella:	http://www.genedb.org/genedb/etenella/
G. lamblia:	*http://giardiadb.org/giardiadb/*
M. domestica	http://genome.ucsc.edu/cgi-bin/hgBlat
P. marinus	*http://pre.ensembl.org/Petromyzon_marinus/index.html*
P. tetraurelia	http://www.genoscope.cns.fr/externe/Francais/Projets/Projet_FN/
Plasmodium sp.	http://www.plasmodb.org/plasmo/home.jsp
T. thermophila:	http://www.ciliate.org/
T. gondii:	http://www.toxodb.org/toxo-release4-0/home.jsp
T. nigroviridis:	http://www.genoscope.cns.fr/externe/tetranew/
T. rubripes:	http://www.fugu-sg.org/project/info.html

Cyanobacteria   http://www.kazusa.or.jp/cyano/cyano.html

Kingdom Plantae databases

Plant species:	http://www.sgn.cornell.edu/tools/blast/index.pl
Plant species:	http://www.plantgdb.org/
Plant species:	http://genome.jgi-psf.org/
C. reinhaardtii	http://www.chlamy.org/
