# Supplementary material for: Conservation of the glucan phosphatase laforin is linked to rates of molecular evolution and the glucan metabolism of the organism
Source: BMC Evol Biol. 2009 Jun 22;9:138. doi: 10.1186/1471-2148-9-138 (PMC2714694; doi:10.1186/1471-2148-9-138)
Supplement: Additional file 2 — Summary of metazoan phylogeny. This is a depiction of an unrooted phylogeny showing the major classifications of metazoans (note: not to scale). Highlighted in yellow are organisms or groups of organisms that contain laforin. Monosiga brevicollis is not a metazoan, but is arguably the closest unicellular relative to metazoans. For a broad frame of reference visit . [file 1471-2148-9-138-S2.pdf]

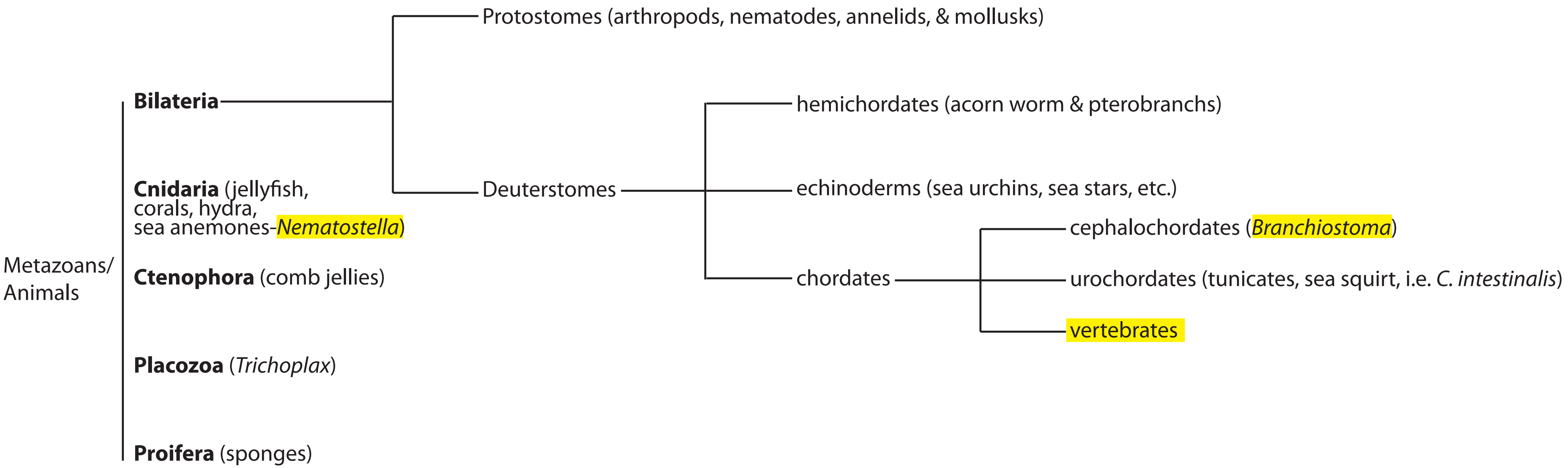

Monosiga brevicollis, a choanoflagellate, is arguably the closest unicellular relative to metazons
