# Supplementary figures and images for: Conservation of the glucan phosphatase laforin is linked to rates of molecular evolution and the glucan metabolism of the organism
Source: BMC Evol Biol. 2009 Jun 22;9:138. doi: 10.1186/1471-2148-9-138 (PMC2714694; doi:10.1186/1471-2148-9-138)

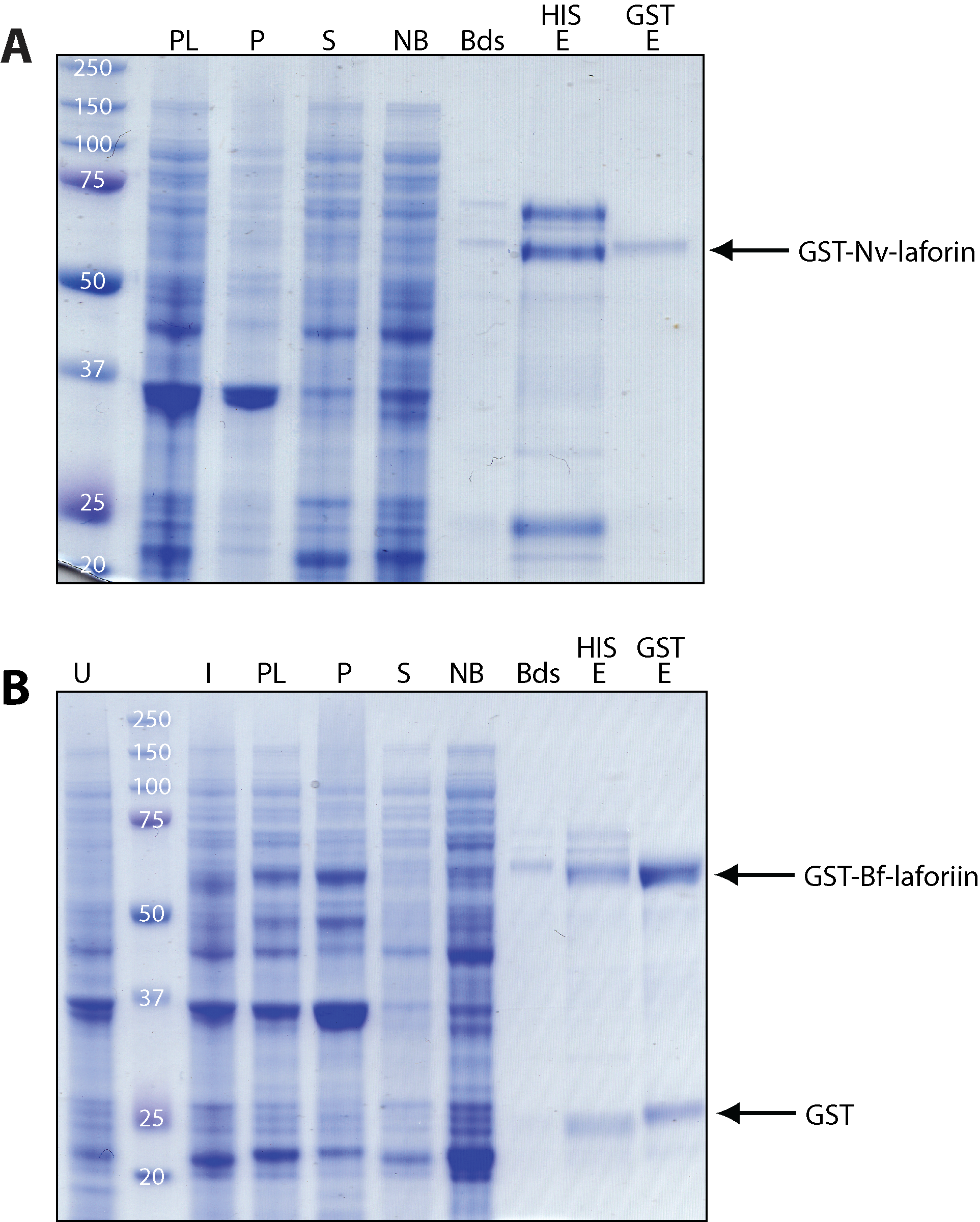

Supplement: Additional file 3 — Purification of recombinant GST-Bf-laforin and GST-Nv-laforin. A, GST-Nv-laforin-HIS6 was purified from soluble E. coli lysate via Ni2+-agarose affinity chromatography. B, GST-Bf-laforin-HIS6 was purified from soluble E. coli lysate via Ni2+-agarose affinity chromatography. U, uninduced; I, induced; PL, post-lysis; P, pellet (insoluble) fraction; S, soluble fraction; NB, not bound to beads; Bds, bound to HIS beads; HIS E, HIS eluate; GST E, GST eluate. [file 1471-2148-9-138-S3.png]

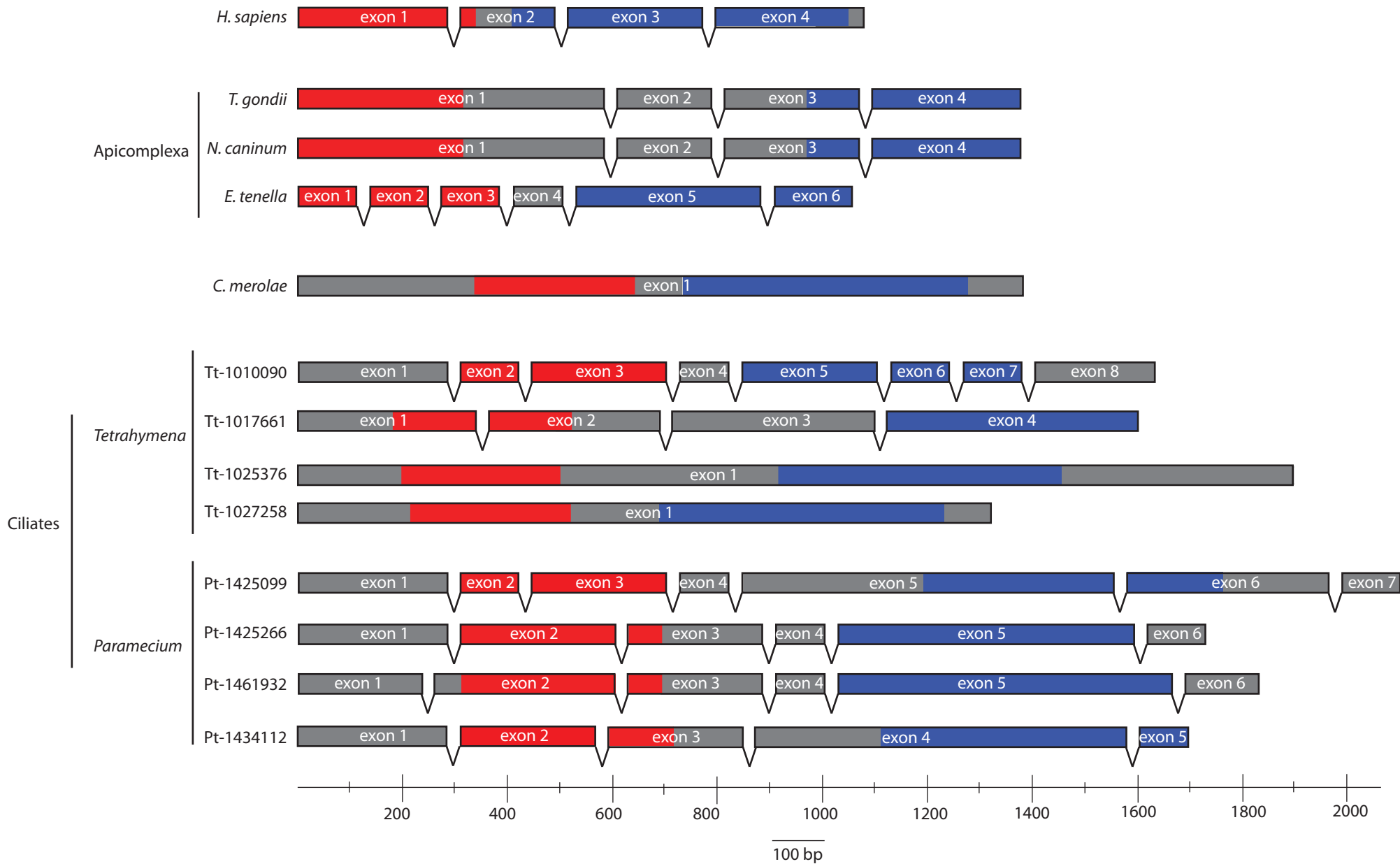

Supplement: Additional file 4 — Intron-exon numbers and boundaries of laforin genes. Predicted intron-exon boundaries for the genes encoding Hs-, Tg-, Nc-, Et-, Cm-, Tt- and Pt-laforin. The coding region that encodes the CBM is highlighted in red, the DSP in blue, and the rest is in gray. Accession numbers are listed in Additional File 8. [file 1471-2148-9-138-S4.pdf]
