# Supplementary material for: Conservation of the glucan phosphatase laforin is linked to rates of molecular evolution and the glucan metabolism of the organism
Source: BMC Evol Biol. 2009 Jun 22;9:138. doi: 10.1186/1471-2148-9-138 (PMC2714694; doi:10.1186/1471-2148-9-138)
Supplement: Additional file 5 — Table of small subunit (SSU) ribosomal RNA (rRNA) accession numbers. Listed on the left are the organisms from the phylogeny in Figure 1 and on the right are the accession numbers for the SSU rRNA genes. Each of the accession numbers are from NCBI Genbank unless otherwise noted. [file 1471-2148-9-138-S5.pdf]

Additional File 5 Small subunit (SSU) ribosomal RNA (rRNA) accession numbers.

A. californica	AY039804
A. pectinifera	AB084551
A. thaliana	X16077
B. floridea	M97571
C. elegans	AY268117
C. intestinalis	AB013017
C. merolae	AB158483
C. paradoxa	NC_001675.1
C. parvum	AF093489
C. reinhardtii	M327083
Capitella sp.	CCU67323
D. japonica	AF013153
D. melanogaster	M21017.1
E. coli	Z83205
E. histolytica	AF149911
E. huxleyi	X82156
E. tenella	U67121
G. gallus	AF173612
G. theta	NC_000926.1
H. echinata	AY920763
H. sapiens	X03205
L. gigantea	AF308645
M. brevicollis	AF100940
Mertensiid sp.	AF293675
N. vectensis	AF254382
Nostoc sp.	NC_003272.1
O. carmela	EU702422
P. falciparum	M19172
P. marinus	FSARREE
P. tetraurelia	X03772
S. cerevisiae	Z75578
S. kowalevskii	AF236801
Trichoplax sp.	Z22783
T. cruzi	AF303660
T. gondii	X68523
T. nigroviridis	chrUn_random:61030224..62171431---- Tetraodon Genome Browser
T. thermophila	X56165
X. laevis	X04025
