# Supplementary material for: Conservation of the glucan phosphatase laforin is linked to rates of molecular evolution and the glucan metabolism of the organism
Source: BMC Evol Biol. 2009 Jun 22;9:138. doi: 10.1186/1471-2148-9-138 (PMC2714694; doi:10.1186/1471-2148-9-138)
Supplement: Additional file 6 — Table of accession numbers for SEX4 orthologs. [file 1471-2148-9-138-S6.pdf]

Additional File 6 Accession numbers for SEX4 orthologs.

Organism	Database	Accession number
A. thaliana	Genbank	AAN28817
Aquilegia sp.	Genbank	DT739859+DT764798
C. reinhaardtii	JGI	149756
C. sinensis	Genbank	CV886681
G. hirsutum	TIGR	TC38280
M. truncatula	Genbank	BG581666+AW689683
N. tabacum	Sol Genomics Network	SGN-U365585
O. sativa	Genbank	ABF93554
P. patens	JGI	175844
Phaseolus	Genbank	CV538569+CB540037+CV534719
P. sitchensis	Genbank	ABK25215
P. vulgaris	Genbank	CV538569+CB540037+CV534719
S. lycopersicum	Genbank	CAC44460
S. tuberosum	Genbank	ABB87109
V. vinifera	Genbank	CAO68845
Z. mays	Genbank	ACF82323
