# Supplementary material for: Conservation of the glucan phosphatase laforin is linked to rates of molecular evolution and the glucan metabolism of the organism
Source: BMC Evol Biol. 2009 Jun 22;9:138. doi: 10.1186/1471-2148-9-138 (PMC2714694; doi:10.1186/1471-2148-9-138)
Supplement: Additional file 7 — Table of genomes investigated for the presence of laforin. The genome of each organism was searched for laforin and/or SEX4 using the appropriate database (Additional File 4). If laforin was absent an extensive literature search was performed on the organism to determine which of the three criteria it lacked: red algal descent, mitochondrion, and/or floridean starch/LBs. If the organism lacked laforin, then at least one of the three criteria that it lacks is presented beside its name. The organism name of genomes containing laforin are bold and in green. The organism name of genomes that are nearing completion and that contain laforin based on our predictions are bold and in red. The phrase "incomplete genome, has laforin" refers to organisms with incomplete genomes, but where a partial CBM and DSP corresponding to laforin was found. Highlighted in yellow are organisms found in this study to contain laforin. Organism classification is based on widely accepted taxonomy classifications [73]. [file 1471-2148-9-138-S7.pdf]

Additional File 7 Genomes investigated for presence of laforin.

I. Eukaryotes

A. <u>Amoebozoa</u>

1. Tubulinea

2. Flabellinea

3. Stereomyxida

4. Acanthamoebidae

5. Entamoebida

Entamoeba dispar	lacks mitochondrion, non-red algal descent
Entamoeba histolytica	lacks mitochondrion, non-red algal descent
Entamoeba invadens	lacks mitochondrion, non-red algal descent
Entamoeba moshkovskii	lacks mitochondrion, non-red algal descent
Entamoeba terrapinae	lacks mitochondrion, non-red algal descent

6. Mastigamoebidae

7. *Pelomyxa*

8. Eumycetozoa

Dictyostelium discoideum	non-red algal descent, fast evolving genome

B. <u>Opisthokonta</u>

1. Fungi

Ajellomyces capsulatus	non-red algal descent, fast evolving genome
Ascosphaera apis	non-red algal descent, fast evolving genome
Ashbya gossypii	non-red algal descent, fast evolving genome
Aspergillus clavatus	non-red algal descent, fast evolving genome
Aspergillus flavus	non-red algal descent, fast evolving genome
Aspergillus fumigatus	non-red algal descent, fast evolving genome
Aspergillus nidulans	non-red algal descent, fast evolving genome
Aspergillus terreus	non-red algal descent, fast evolving genome
Batrachochytrium dendrobatidis	non-red algal descent, fast evolving genome
Botryotinia fuckelinana	non-red algal descent, fast evolving genome
Candida albicans	non-red algal descent, fast evolving genome
Candida glbrata	non-red algal descent, fast evolving genome
Candida tropicalis	non-red algal descent, fast evolving genome
Chaetomium globosum	non-red algal descent, fast evolving genome
Clavispora lusitaniae	non-red algal descent, fast evolving genome
Coccidioides immitis	non-red algal descent, fast evolving genome
Coccidioides posadasii	non-red algal descent, fast evolving genome
Coprinopsis cinerea okayama	non-red algal descent, fast evolving genome
Cryptococcus neoformans sp.	non-red algal descent, fast evolving genome
Debaryomyces hansenii	non-red algal descent, fast evolving genome
Encephalitozoon cuniculi	non-red algal descent, fast evolving genome

Eremothecium gossypii	non-red algal descent, fast evolving genome
Fusarium oxysporum	non-red algal descent, fast evolving genome
Gibberella monoiliformis	non-red algal descent, fast evolving genome
Gibberella zeae	non-red algal descent, fast evolving genome
Kluyveromyces lactis	non-red algal descent, fast evolving genome
Kluyveromyces waltii	non-red algal descent, fast evolving genome
Laccaria bicolor	non-red algal descent, fast evolving genome
Lodderomyces elongisporus	non-red algal descent, fast evolving genome
Magnaporthe grisea	non-red algal descent, fast evolving genome
Malassezia furfur	non-red algal descent, fast evolving genome
Mycosphaerella graminocola	non-red algal descent, fast evolving genome
Neosartorya fischeri	non-red algal descent, fast evolving genome
Nectria haematococca	non-red algal descent, fast evolving genome
Neurospora crassa	non-red algal descent, fast evolving genome
Phaeosphaeria nodorum	non-red algal descent, fast evolving genome
Phanerochaete chrysosporium	non-red algal descent, fast evolving genome
Pichia guilliermondii	non-red algal descent, fast evolving genome
Pichia stipitis	non-red algal descent, fast evolving genome
Penicillium marneffei	non-red algal descent, fast evolving genome
Pneumocystis carnii	non-red algal descent, fast evolving genome
Podospora anserina	non-red algal descent, fast evolving genome
Puccinia graminis	non-red algal descent, fast evolving genome
Pyrenophora tritici-repentis	non-red algal descent, fast evolving genome
Rhizopus oryzae	non-red algal descent, fast evolving genome
Saccharomyces bayanus	non-red algal descent, fast evolving genome
Saccharomyces castellii	non-red algal descent, fast evolving genome
Saccharomyces cerevisiae	non-red algal descent, fast evolving genome
Saccharomyces kluyveri	non-red algal descent, fast evolving genome
Saccharomyces kudriavzevii	non-red algal descent, fast evolving genome
Saccharomyces mikatae	non-red algal descent, fast evolving genome
Saccharomyces paradoxus	non-red algal descent, fast evolving genome
Schizosaccharomyces japonicus	non-red algal descent, fast evolving genome
Schizosaccharomyces pombe	non-red algal descent, fast evolving genome
Sclerotinia sclerotiorum	non-red algal descent, fast evolving genome
Sporobolomyces roseus	non-red algal descent, fast evolving genome
Talaromyces stipitatus	non-red algal descent, fast evolving genome
Trichoderma atroviride	non-red algal descent, fast evolving genome
Trichoderma reesei	non-red algal descent, fast evolving genome
Trichoderma virens	non-red algal descent, fast evolving genome
Uncinocarpus reesii	non-red algal descent, fast evolving genome
Ustilago maydis	non-red algal descent, fast evolving genome
Vanderwaltozyma polyspora	non-red algal descent, fast evolving genome
Yarrowia lipolytica	non-red algal descent, fast evolving genome

2. Mesomycetozoa

3. Choanomonada

Monosiga brevicollis	fast evolving genome

4. Metazoa

Capitella sp.	annelid, fast evolving genome
Helobdella robusta	annelid, fast evolving genome
Aedes aegypti	arthropod, fast evolving genome
Anopheles gambiae	arthropod, fast evolving genome
Apis mellifera	arthropod, fast evolving genome
Bombyx mori	arthropod, fast evolving genome
Culex pipens	arthropod, fast evolving genome
Daphnia pulex	arthropod, fast evolving genome
Drosophila ananassae	arthropod, fast evolving genome
Drosophila erecta	arthropod, fast evolving genome
Drosophila grimshawi	arthropod, fast evolving genome
Drosophila mojavensis	arthropod, fast evolving genome
Drosophila persimilis	arthropod, fast evolving genome
Drosophila pseudoobscura	arthropod, fast evolving genome
Drosophila sechellia	arthropod, fast evolving genome
Drosophila simulans	arthropod, fast evolving genome
Drosophila virilis	arthropod, fast evolving genome
Drosophila willistoni	arthropod, fast evolving genome
Drosophila yakuba	arthropod, fast evolving genome
Glossina morsitans	arthropod, fast evolving genome
Nasonia vitripennis	arthropod, fast evolving genome
Pediculus humanus corporis	arthropod, fast evolving genome
Tribolium castaneum	arthropod, fast evolving genome
Branchiostoma floridea	**cephlachordate, has laforin**
Hydra sp.	cnidarian, fast evolving genome
Nematostella vectensis	**cnidarian, has laforin**
Lottia gigantea	mollusk, fast evolving genome
Ascaris lumbicoides	nematode, fast evolving genome
Brugia malayi	nematode, fast evolving genome
Caenorhabditis briggsae	nematode, fast evolving genome
Caenorhabditis brenneri	nematode, fast evolving genome
Caenorhabditis elegans	nematode, fast evolving genome
Caenorhabditis remanei	nematode, fast evolving genome
Haemonchus contortus	nematode, fast evolving genome
Trichoplax sp.	placozoa, fast evolving genome

Fasciola hepatica	platyhelminthes, fast evolving genome
Schistosoma mansoni	platyhelminthes, fast evolving genome
Aplysia californica	urochordate, fast evolving genome
Ciona intestinalis	urochordate, fast evolving genome
Ciona savignyi	urochordate, fast evolving genome
Strongylocentrotus purpuratus	urochordate, fast evolving genome
**vertebrates**	
Anolis carolinensis	**reptile, incomplete genome, has laforin**
Bos taurus	**mammal, has laforin**
Canis familiaris	**mammal, has laforin**
Cavia porcellus	**mammal, incomplete genome, has laforin**
Danio rerio	**osteichthyes, has laforin**
Dasypus novemcinctus	**mammal, incomplete genome, has laforin**
Echinops telfairi	**mammal, incomplete genome, has laforin**
Equus caballus	**mammal, incomplete genome, has laforin**
Erinaceus eruopaeus	**mammal, incomplete genome, has laforin**
Felis catus	**mammal, has laforin**
Gallus gallus	**aves, has laforin**
Gasterosteus aculeatus	**osteichthyes, has laforin**
Gorilla gorilla	**mammal, incomplete genome, has laforin**
Homo sapiens	**mammal, has laforin**
Loxodonta africana	**mammal, has laforin**
Macaca mulatta	**mammal, has laforin**
Microcebus murinus	**mammal, incomplete genome, has laforin**
Monodelphis domestica	**mammal, has laforin**
Mus musculus	**mammal, has laforin**
Myotis lucifugus	**mammal, incomplete genome, has laforin**
Ornithorhynchus anatinus	**mammal, has laforin**
Oryctolagus cuniculus	**mammal, incomplete genome, has laforin**
Oryzias latipes	**osteichthyes, has laforin**
Otolemur garnettii	**mammal, incomplete genome, has laforin**
Pan troglodytes	**mammal, has laforin**
Pongo pygmaeus	**mammal, incomplete genome, has laforin**
Rattus norvegicus	**mammal, has laforin**
Sorex araneus	**mammal, incomplete genome, has laforin**
Sus scrofa	**mammal, has laforin**
Takifugu rubripes	**osteichthyes, has laforin**
Tetraodon nigroviridis	**osteichthyes, has laforin**
Xenopus laevis	**amphibian, has laforin**

C. <u>Rhizaria</u>

1. Cercozoa

Bigelowiella natans	plant pathogen, lacks floridean starch
Phytophthora infestans	plant pathogen, fast evolving genome
Phytophthora ramorum	plant pathogen, fast evolving genome
Phytophthora sojae	plant pathogen, fast evolving genome

2. Haplosporidia

3. Foraminifera

4. *Gromia*

5. Radiolaria

D. <u>Archaeplastida</u>

1. Glaucophyta

Cyanophora paradoxa	algae, not of red algal descent

2. Rhodophyceae

Cyanidioschyzon merolae	**has laforin**
Galdieria sulphuraria	**incomplete genome, likely has laforin**

3. Chloroplastida

Arabidopsis thaliana	land plant, has SEX4
Aquilegia sp.	land plant, has SEX4
Chlamydomonas reinhardtii	green alga, has SEX4
Citrus sinensis	land plant, has SEX4
Medicago truncatula	land plant, has SEX4
Micromonas sp.	phytoplankton, has SEX4
Oryza sativa	land plant, has SEX4
Osterococcus lucimarinus	green alga, has SEX4
Ostreococcus tauri	green alga, has SEX4
Phaseolus vulgaris	land plant, has SEX4
Physcomitrella patens	moss, has SEX4
Selaginella moellendorffii	land plant, has SEX4
Solanum lycopersicum	land plant, has SEX4
Solanum tuberosum	land plant, has SEX4
Sorghum bicolor	land plant, has SEX4
Triticum aestivum	land plant, has SEX4
Vitis vinifera	land plant, incomplete genome, has SEX4
Volvox carteri	green alga, has SEX4
Zea mays	land plant, has SEX4

E. Chromalveolata

1.Cryptophyceae

Guillardia theta	**nucleomorph sequenced, nuclear genome not sequenced, likely has laforin**
Hemiselmis andersenii	**nucleomorph sequenced, nuclear genome not sequenced, likely has laforin**

2. Haptophyta

Emiliania huxleyi	phytoplankton, fast evolving genome

3. Stramenopiles

Hyaloperonospora parasitica	water mold, lacks floridean starch
Thalassiosira pseudonana	diatom, fast evolving genome
Phaeodactylum tricornutum	diatom, fast evolving genome

4. Alveolata

Babesia bovis	lacks floridean starch
Babesia bigemina	lacks floridean starch
Babesia malayi	lacks floridean starch
Cryptosporidium parvum	lacks mitochondrion
Cryptosporidium hominis	lacks mitochondrion
Eimeria tenella	**has laforin**
Neospora caninum	**has laforin**
Paramecium tetraurelia	**has laforin**
Perkinsus marinus	lacks floridean starch
Plasmodium berghei	lacks floridean starch
Plasmodium chabaudi	lacks floridean starch
Plasmodium falciparum	lacks floridean starch
Plasmodium gallinaceum	lacks floridean starch
Plasmodium knowlesi	lacks floridean starch
Plasmodium reichenowi	lacks floridean starch
Plasmodium vivax	lacks floridean starch
Plasmodium yhoelii yoelii	lacks floridean starch
Sarcocystis neurona	**incomplete genome, likely has laforin**
Theileria annulata	lacks floridean starch
Theileria parva	lacks floridean starch
Tetrahymena thermophila	**has laforin**
Toxoplasma gondii	**has laforin**

F. Excavata

1. Fornicata

Giardia lamblia	lacks mitochondrion, non-red algal descent

2. *Malawimonas*

3. Parabasalia

Trichomonas vaginalis	lacks mitochondrion, non-red algal descent

4. Preaxostyla

5. Jakobida

6. Heterolobosea

Naegleria gruberi	non-red algal descent

7. Euglenozoa

Crithidia deanei	non-red algal descent

Leishmania braziliensis	non-red algal descent
Leishmania infantum	non-red algal descent
Leishmania major	non-red algal descent
Leptomonas seymouri	non-red algal descent
Trypanosoma brucei	non-red algal descent
Trypanosoma congolense	non-red algal descent
Trypanosoma cruzi	non-red algal descent
Trypanosoma gambiense	non-red algal descent

II. Prokaryotes--Archaea and Eubacteria

All 940 microbial genomes in NCBI	lack mitochondrion, no floridean starch, non-red algal descent, and fast evolving genome
