# Supplementary material for: Conservation of the glucan phosphatase laforin is linked to rates of molecular evolution and the glucan metabolism of the organism
Source: BMC Evol Biol. 2009 Jun 22;9:138. doi: 10.1186/1471-2148-9-138 (PMC2714694; doi:10.1186/1471-2148-9-138)
Supplement: Additional file 8 — Table of accession numbers for laforin orthologs. [file 1471-2148-9-138-S8.pdf]

Additional File 8 Accession numbers for laforin orthologs.

Organism	Database	Accession number
B. floridae-264	Genbank	EEA65811
	JGI protein ID	264224
B. floridae-201	Genbank	EEA77678
	JGI protein ID	201927
B. floridae-743	Genbank	EEA68580
	JGI protein ID	74326
B. taurus	Genbank	XP_605269
C. merolae	*C. merolae* Genome Project	CMT465C
C. familiaris	Genbank	XP_541139
D. rerio	Genbank	XP_688154
E. tenella	GeneDB	Et_v1_Twnscn_Contig6817.tmp13
G. gallus	Genbank	NP_001026240
H. sapiens	Genbank	NP_005661
L. africana	Ensmbl	ENSLAFP00000008065
M. domestica	Genbank	XP_001381051
M. mulatta	Genbank	XP_001087462
M. musculus	Genbank	NP_034276
N. caninum	GeneDB	NC_LIV_081160
N. vectensis	Genbank	XP_001624235
O. anatinus	Genbank	XP_001506932
P. tetraurelia	Genbank	XP_001425099
P. tetraurelia	Genbank	XP_001425266
P. tetraurelia	Genbank	XP_001461932
P. tetraurelia	Genbank	XP_001434112
P. troglodytes	Genbank	XP_001161292
R. norvegicus	Genbank	XP_001070057
S. scrofa	Genbank	XP_001927667
T. gondii	Genbank	EEB00621
T. nigroviridis	Genbank	CAG03589
T. thermophila	Genbank	EAR89845
T. thermophila	Genbank	XP_001010090
T. thermophila	Genbank	XP_001017661
T. thermophila	Genbank	XP_001025376
T. thermophila	Genbank	XP_001027258
X. laevis	Genbank	AAH73202
